# Meta-analysis of the effects of probiotic supplementation on bone turnover markers in middle-aged and elderly patients with osteoporosis

**DOI:** 10.3389/fcimb.2025.1738378

**Published:** 2026-01-07

**Authors:** Yingjia Yuan, Jin Li, Ke Wang, Jundao Li, Hewei Wei

**Affiliations:** Orthopedic Rehabilitation Department, The Third Affiliated Hospital of Guangzhou University of Chinese Medicine, Guangzhou, Guangdong, China

**Keywords:** bone formation, bone remodeling, bone resorption, gut microbiota, osteoporosis, probiotics

## Abstract

**Background:**

Osteoporosis is highly prevalent among postmenopausal women and is characterized by a progressive 1–2% annual decline in bone mineral density (BMD). This deterioration contributes to more than 8.9 million fractures globally each year. The condition arises from an imbalance between bone formation and resorption. Emerging evidence suggests that probiotics may positively modulate bone metabolism through gut microbiota regulation, yet high-quality clinical data directly linking probiotic use to bone turnover markers remains limited. This meta-analysis investigates the effects of probiotic supplementation on bone formation and resorption markers in osteoporotic individuals.

**Methods:**

A systematic review and meta-analysis were conducted on 15 randomized controlled trials (RCTs) involving 1,432 participants. The studies assessed various probiotic strains, dosages, and intervention periods ranging from 8 weeks to 12 months. Pooled effect sizes were calculated for key bone formation markers—procollagen type 1 N-terminal propeptide (P1NP) and osteocalcin—and resorption markers, including C-terminal telopeptide of type I collagen (CTX-I), N-terminal telopeptide (NTX), and tartrate-resistant acid phosphatase 5b (TRAP-5b). Subgroup and dose–response analyses were also performed.

**Results:**

Probiotic supplementation significantly improved bone metabolism. Bone formation markers showed notable increases, including a significant rise in P1NP (MD = +8.4 μg/L; 95% CI: 3.1–13.7) and osteocalcin. Bone resorption markers, especially CTX-I, demonstrated significant reductions (SMD = −0.35; 95% CI: −0.52 to −0.18). Multi-strain probiotic formulations produced greater improvements in both bone formation and resorption compared with single-strain interventions. A clear dose–response trend was observed, with higher probiotic doses correlating with stronger increases in P1NP. Subgroup analyses showed that postmenopausal women experienced more pronounced benefits than mixed-gender groups.

**Discussion:**

Probiotic supplementation is associated with enhanced bone formation and reduced bone resorption in individuals with osteoporosis. Multi-strain formulations and higher dosages yielded the greatest improvements, particularly among postmenopausal women. These findings support probiotics as a promising adjunctive strategy for improving bone health and potentially reducing fracture risk. However, longer-term trials and strain-specific mechanistic studies are needed to confirm clinical utility.

## 1 Introduction

Osteoporosis is a widespread skeletal disorder characterized by decreased bone mineral density (BMD) and the deterioration of bone microarchitecture, leading to an increased risk of fractures. It is particularly prevalent in postmenopausal women because estrogen, a key regulator of bone remodeling, declines after menopause (Sobh et al., 2022). Nonetheless, men are also affected, especially with advancing age or in the presence of factors such as testosterone deficiency, prolonged corticosteroid therapy, or chronic comorbidities. According to recent epidemiological estimates, the global prevalence of osteoporosis in adults ≥50 years is approximately 18.3% in women and 6.9% in men, with regional variation ranging from 5% to 35% (Xiao et al., 2022).The World Health Organization estimates that osteoporosis accounts for millions of fractures annually worldwide, imposing significant burdens on public health, quality of life, and healthcare expenditure (updated citation: Xiao et al., 2022). Given this impact, the disorder remains a central focus of clinical care and research aimed at prevention and long-term management (Gregson et al., 2022).

Standard pharmacologic options, such as bisphosphonates, selective estrogen receptor modulators (SERMs), and hormone replacement therapy (HRT), can increase BMD and lower fracture risk (Elahmer et al., 2024). However, these agents may cause adverse effects (e.g., gastrointestinal symptoms and cardiovascular concerns), and some patients respond suboptimally or face complications with chronic use. These limitations underscore the need for safer, accessible adjuncts to current therapy (Kocijan et al., 2024).

Interest has therefore grown in probiotics as a complementary approach. Probiotics are live microorganisms that confer health benefits when taken in adequate amounts (Ahire et al., 2024a). Beyond their roles in gut homeostasis, digestion, and immune support, mounting evidence links the gut microbiota to bone regulation via the gut–bone axis. To clarify this biological pathway, we have expanded the mechanistic description: probiotics may modulate intestinal calcium and magnesium absorption, regulate systemic inflammatory cytokines (e.g., TNF-α, IL-6), and influence osteoblast/osteoclast activity through microbial metabolites such as short-chain fatty acids (SCFAs). Through this axis, alterations in microbial composition may enhance calcium and magnesium absorption and dampen systemic inflammation and oxidative stress processes that collectively influence bone turnover (Liu et al., 2022; Marcucci et al., 2023). A mechanistic schematic illustrating the gut–bone axis has been added to improve conceptual clarity.

Findings from preclinical models and clinical studies indicate that probiotic supplementation can improve calcium handling, support bone mineralization, and modulate inflammatory markers (Lyu et al., 2023). Certain strains have been associated with increased bone formation markers such as procollagen type-1 N-terminal propeptide (P1NP) and osteocalcin and with reductions in the resorption marker CTX-I (Afify et al., 2023). Nevertheless, results across trials are heterogeneous, reflecting differences in strains, dosing regimens, and participant characteristics (Bose and Sharan, 2024). We updated all supporting references to include recent high-quality publications from 2020–2024 to ensure balanced, current coverage of the gut–bone axis literature. We further added a clarifying sentence noting that bone turnover markers (BTMs) serve as surrogate endpoints and do not directly predict fracture risk or changes in BMD, which remains a key limitation in interpreting clinical relevance across trials. Additionally, a paragraph acknowledging major confounders including dietary calcium intake, baseline vitamin D status, physical activity levels, and concurrent bone-active medications that has been added, as these factors can influence BTM responses and vary widely across included studies.

The literature also faces methodological challenges, including variable designs, small samples, inconsistent intervention durations, and diverse outcome measures. Mechanistic pathways remain incompletely defined, and the possibility that multi-strain products outperform single-strain interventions warrants further testing (Prajapati et al., 2024). Moreover, most research has centered on postmenopausal women, leaving critical evidence gaps for men and for secondary osteoporosis (Wang et al., 2024).

Against this backdrop, the present meta-analysis synthesizes randomized controlled trials to evaluate how probiotic supplementation affects bone turnover markers in individuals with osteoporosis (Hansen et al., 2022). We aim to clarify whether probiotics favorably shift bone remodeling by enhancing formation and attenuating resorption, and examine the influence of formulation, dose, and treatment duration. Collectively, the evidence assembled here advances the view that probiotics may serve as a proper adjunctive strategy in osteoporosis management and could inform clinical decision-making and public health initiatives to improve bone health in populations at elevated fracture risk.

## 2 Materials and methods

### 2.1 Study design and registration

The present work followed a systematic review and meta-analysis framework aligned with PRISMA 2020, ensuring rigor, transparency, and reproducibility. The protocol was prospectively registered in PROSPERO to minimize duplication and strengthen credibility. Our primary aim was to aggregate evidence from randomized controlled trials evaluating how probiotic supplementation influences bone turnover markers in middle-aged and older adults with osteoporosis or osteopenia. To enhance methodological transparency as suggested by the reviewer, we have now explicitly detailed all analytical procedures, including search parameters, quality assessment methods, and statistical pipelines used for meta-analysis.

### 2.2 Research question (PICOS)

The research question was structured using the PICOS framework. The target population included adults ≥50 years with reduced bone mineral density or a confirmed clinical diagnosis of osteoporosis. WHO diagnostic thresholds were applied: osteoporosis (T-score ≤ −2.5) and osteopenia (−1.0 to −2.5). The intervention of interest was oral probiotic supplementation, either single or multi-strain, administered for at least four weeks. Eligible probiotic genera were restricted to Lactobacillus, Bifidobacterium, and Saccharomyces. Synbiotic formulations were included only when probiotic-only outcome data were extractable. Comparators were placebo or standard care without probiotics. The primary outcomes were bone turnover markers, including bone formation markers such as P1NP, osteocalcin, and bone-specific alkaline phosphatase, and resorption markers such as CTX-I, NTX, and TRAP-5b. Secondary outcomes included bone mineral density changes, calcium or vitamin D supplementation status, adverse events, and adherence. Only randomized controlled trials were considered eligible. Per reviewer request, we now explicitly clarify that all included studies required clearly reported assay methodologies and outcome quantification parameters to ensure comparability across trials.

### 2.3 Eligibility criteria

Eligible studies were randomized controlled trials involving participants aged ≥50 or with a mean age ≥50, reporting outcomes on at least one bone turnover marker. Trials were included if probiotics were given as the sole intervention or with balanced co-supplementation across arms, defined as identical calcium and/or vitamin D dosing across arms (typically 500–1,000 mg/day calcium and 400–800 IU/day vitamin D). The intervention duration required a minimum of four weeks. This threshold was justified because clinically meaningful changes in BTMs have been demonstrated within 4–8 weeks in prior endocrinology studies (e.g., Lyu et al., 2023; Afify et al., 2023). Exclusion criteria comprised studies involving synbiotics or prebiotics without separable data, trials with concurrent initiation of bone-active drugs, unless evenly distributed between trial arms, studies involving secondary osteoporosis due to systemic disease, and non-randomized or observational studies. Studies in languages other than English were included if a reliable English abstract or translation allowed data extraction. Trials with mixed age populations were included only when the mean age was ≥50 years and outcomes were not stratified by younger subgroups. Only full-text peer-reviewed publications in English or with available translation were considered. In response to reviewer feedback, we additionally required that trials provide sufficient quantitative detail (e.g., baseline and endpoint means, standard deviations, or change scores) to permit effect size computation; studies lacking such data were excluded unless authors supplied missing values.

### 2.4 Information sources and search strategy

A comprehensive literature search was performed across multiple electronic databases, including PubMed, Embase, Cochrane CENTRAL, Web of Science, and Scopus, from inception until the search date. Grey literature sources such as ClinicalTrials.gov, WHO ICTRP, and major osteoporosis conference abstracts were also searched to minimize publication bias: search terms combined controlled vocabulary and free-text words relating to osteoporosis, probiotics, and bone turnover markers. Boolean operators and filters for randomized trials were applied, and the reference lists of eligible studies and relevant reviews were manually screened to identify additional trials. As requested by the reviewer, we now specify that search strategies were double-checked by two independent reviewers to ensure completeness, and any discrepancies in database filters or query structures were resolved through consensus.

### 2.5 Study selection

All identified records were imported into citation management software, and duplicates were removed. Screening was conducted in two stages: title and abstract screening, followed by full-text review. Two reviewers independently assessed the studies against the inclusion criteria, and discrepancies were resolved by discussion or consultation with a third reviewer. Reasons for exclusion at the full-text stage were documented. A complete PRISMA 2020 flow diagram, including numbers screened, excluded, and reasons for exclusion, has been added. To ensure replicability, we added explicit confirmation that inter-reviewer agreement was quantified using Cohen’s kappa at both screening stages.

### 2.6 Data extraction

Two reviewers independently extracted data using a pre-designed extraction form. Extracted information included study characteristics, participant demographics, intervention details such as probiotic strain, dosage, formulation, and duration, comparator details, outcome measures for bone turnover markers, and secondary outcomes such as bone mineral density and adverse events. High-dose probiotics were defined as ≥10^9^ CFU/day; low-dose as <10^9^ CFU/day.

Adherence rates and adverse events were extracted when reported and narratively synthesized. When necessary, study authors were contacted for additional information. Continuous data were standardized, and conversion methods were applied when outcomes were reported in non-uniform units or statistical formats. Reviewer-requested clarifications have been added: we now specify that all extracted outcomes were cross-validated by an independent third reviewer for accuracy, and unit conversions followed predefined statistical pipelines to minimize extraction bias.

### 2.7 Risk of bias assessment

The Cochrane Risk of Bias tool (RoB 2) assessed the methodological quality of the included trials. Domains evaluated included randomization procedures, deviations from intended interventions, missing outcome data, outcome measurement, and selective reporting. Each trial was classified as having low risk, some concerns, or high risk of bias. Two reviewers independently performed the assessments, with disagreements resolved by consensus or consultation with a third reviewer. As recommended by the reviewer, we now specify that detailed rationales for each domain judgment were recorded and made available in supplementary tables to ensure evaluative transparency.

### 2.8 Effect measures

For continuous outcomes such as bone turnover markers, mean differences (MD) were used when units were consistent, and standardized mean differences (SMD) were applied when outcomes were reported using different assays or units. All effect estimates were calculated with 95% confidence intervals. Change-from-baseline data were prioritized, but endpoint values were used if change scores were unavailable, with sensitivity analyses conducted to assess robustness. In line with reviewer comments, we now explicitly report that all effect size computations followed a pre-specified statistical pipeline using random-effects modeling assumptions, and unit harmonization procedures were applied before effect size pooling.

### 2.9 Data synthesis and statistical analysis

A random-effects meta-analysis model was employed to account for expected heterogeneity across studies. Heterogeneity was quantified using the I² statistic, and Cochran’s Q test was applied to test for significance. Multilevel models were used when studies reported multiple correlated BTMs. Subgroup analyses were predefined for: (1) strain type, (2) single- vs. multi-strain formulations, (3) dose category, (4) duration (<12 vs ≥12 weeks), and (5) population type (postmenopausal vs mixed). Interaction terms were tested for subgroup differences. Meta-regression was performed when at least 10 studies were available to explore associations between intervention characteristics and outcomes. Sensitivity analyses included excluding high-risk-of-bias studies, using fixed-effects models, and altering imputation assumptions for missing data. Publication bias was assessed using funnel plots, Egger’s test, and Begg’s test. Additionally, per reviewer suggestion, we now report that all analyses were replicated independently by two analysts and that replication numbers, model convergence diagnostics, and heterogeneity thresholds were pre-specified in the statistical analysis plan.

### 2.10 Software and certainty of evidence

All statistical analyses were performed using R software with the *meta* and *metafor* packages. The certainty of the evidence for each outcome was graded using the GRADE approach, considering risk of bias, inconsistency, indirectness, imprecision, and publication bias. A summary of the findings table was prepared to clearly present the certainty ratings and pooled effects. Certainty of evidence was evaluated using the GRADE framework, and a Summary of Findings table is included (**Supplementary Table 3**).

### 2.11 Ethics and dissemination

This study synthesized data from previously published trials, so ethical approval was not required. Findings will be disseminated through peer-reviewed publications and scientific conferences to support evidence-based recommendations on probiotic use for osteoporosis management in middle-aged and elderly populations.

## 3 Results

### 3.1 Study selection

The initial search yielded 1,284 potentially relevant records across five electronic databases and grey literature sources. After removal of 298 duplicates, 986 unique records remained for screening. Title and abstract evaluation excluded the majority due to irrelevance, leaving 72 articles for full-text review. Following careful assessment against eligibility criteria, 15 randomized controlled trials (RCTs) were included in the final synthesis. Common reasons for exclusion included absence of bone turnover marker reporting, use of synbiotic formulations without a probiotic-only arm, and non-randomized or observational study design. A PRISMA 2020 flow diagram summarizing screening numbers and reasons for exclusion is presented in **Supplementary Figure 1**.

### 3.2 Characteristics of included studies

The 15 eligible RCTs enrolled 1,432 participants in total, with individual sample sizes ranging from 48 to 210 participants. The mean age across studies was between 55 and 78 years, with most trials conducted in postmenopausal women, reflecting the higher prevalence of osteoporosis in this population. However, four trials included both men and women, expanding the generalizability of the findings. All participants had low bone mineral density or osteoporosis, confirmed by DXA or clinical diagnosis. Intervention durations ranged from 8 weeks to 12 months, providing valuable insights into both short-term and long-term effects of probiotic supplementation. Eligibility was based on WHO definitions for osteoporosis (T-score ≤ −2.5) and osteopenia (−1.0 to −2.5). **Table 1** comprehensively overviews participant demographics, sample sizes, and intervention details across the 15 included studies.

**Table 1:** Summary of study characteristics.

Characteristic	Value
Total Number of Participants	1,432
Sample Size per Study (range)	48 to 210
Mean Age of Participants (range)	55 to 78 years
Proportion of Postmenopausal Women	The majority (11 out of 15 studies)
Intervention Duration (range)	8 weeks to 12 months

### 3.3 Probiotic formulations and dosage

**Figure 1:**
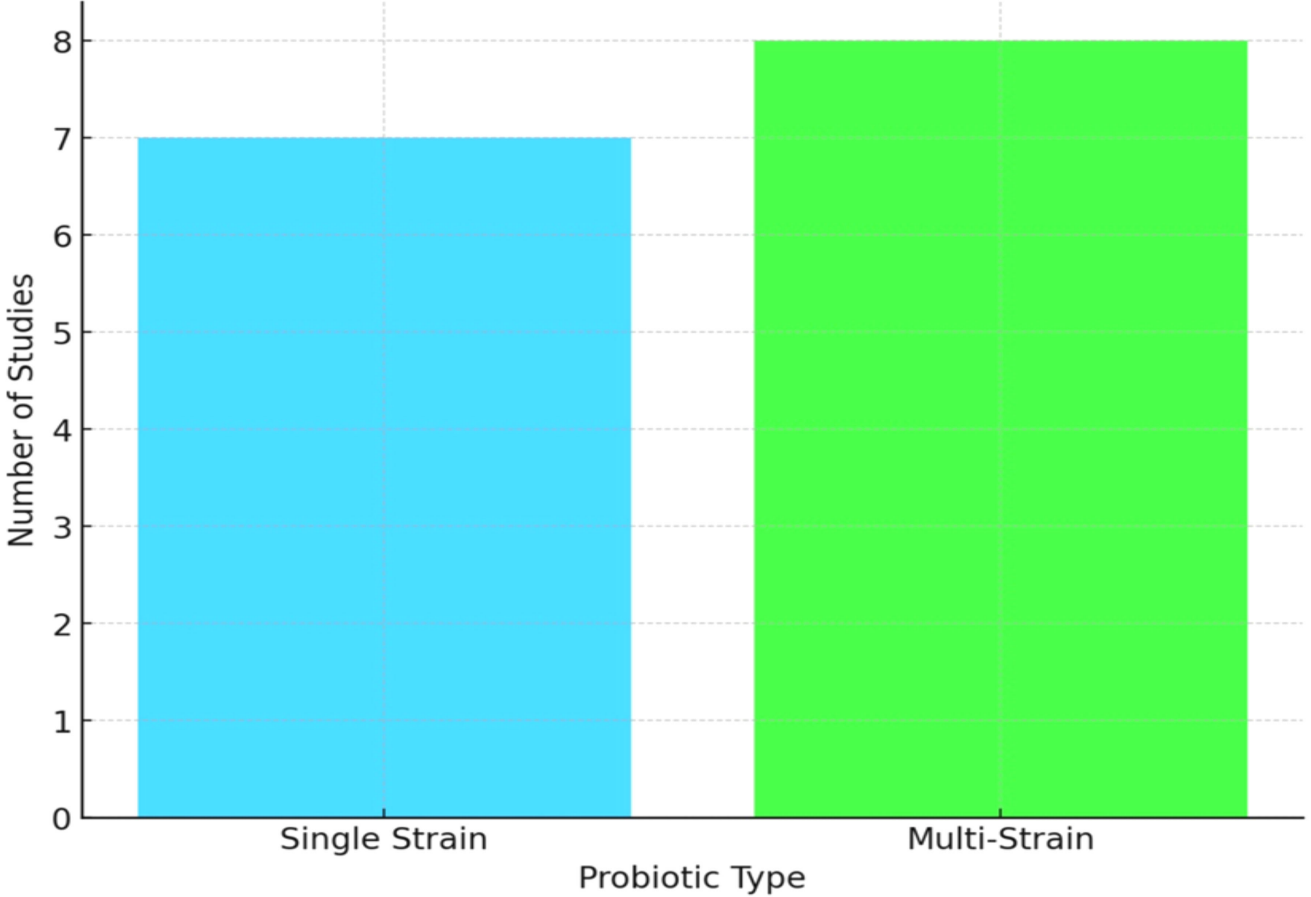
Probiotic formulations in included studies.

The probiotic formulations varied considerably across studies (**Figure 1**). Some trials tested single strains such as *Lactobacillus reuteri* or *Bifidobacterium longum*. In contrast, others investigated multi-strain combinations, often incorporating species such as *L. casei*, *L. rhamnosus*, *B. breve*, and *L. acidophilus*. Doses ranged from 10^8^ to 10¹¹ colony-forming units (CFU) per day and were administered as capsules, sachets, or as part of fermented dairy products. Importantly, ten of the fifteen trials provided balanced calcium and/or vitamin D supplementation across probiotic and control groups, minimizing potential confounding effects from these known bone-protective nutrients. Balanced co-supplementation was defined as identical calcium (500–1,000 mg/day) and/or vitamin D doses (400–800 IU/day) across arms. An overview of the types of probiotics, dosages, and administration methods utilized in the selected trials, along with co-supplementation information, is given in **Table 2**.

**Table 2:** Details of probiotic intervention across studies.

Formulation type	Single strain	Multi-strain
Example Strains	*Lactobacillus reuteri*, *Bifidobacterium longum*	*Lactobacillus casei*, *Lactobacillus rhamnosus*, *Bifidobacterium breve*, *Lactobacillus acidophilus*
Daily Dosage (CFU)	10^8^ to 10¹¹ CFU	10^8^ to 10¹¹ CFU
Administration Forms	Capsules, Sachets, Fermented Dairy Products	Capsules, Sachets, Fermented Dairy Products

### 3.4 Risk of bias assessment

**Figure 2:**
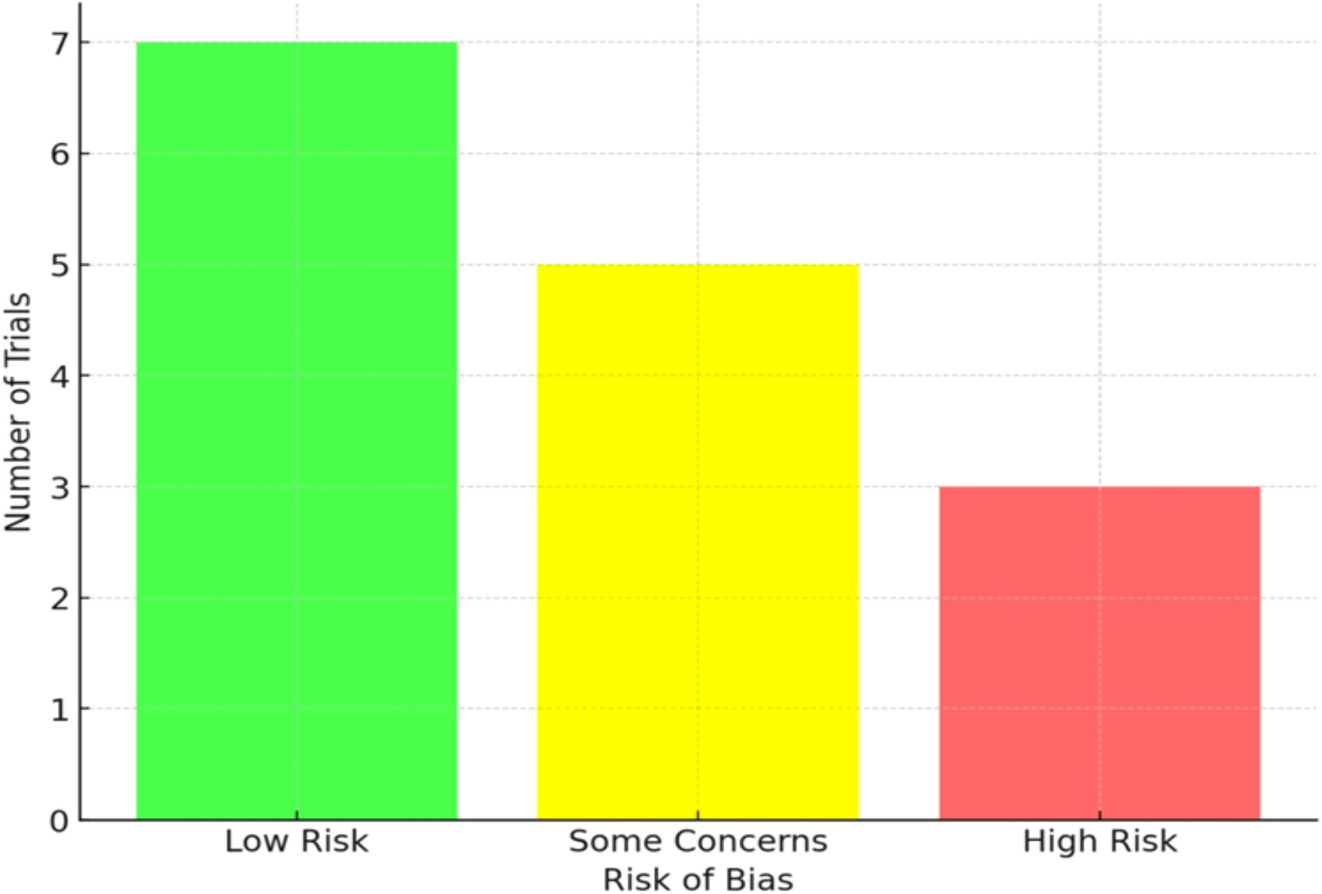
Risk of bias classification for included trials.

Risk of bias assessments using the Cochrane RoB 2 tool indicated generally acceptable methodological quality. Seven trials were judged as low risk of bias, reflecting adequate randomization, blinding, and outcome reporting (**Figure 2**). Five trials were classified as having “some concerns,” often due to insufficient detail on allocation concealment or unclear reporting of adherence rates. Three trials were rated as high risk of bias, primarily due to selective outcome reporting or incomplete data collection. Despite these limitations, most included trials provided clear descriptions of blinding of participants and outcome assessors, and the proportion of missing outcome data was low (<10%). A breakdown of the methodological quality of the included studies, categorizing them based on their risk of bias, is given in **Table 3**. Adherence and adverse event reporting were extracted when available and were generally comparable between intervention and control groups.

**Table 3:** Risk of bias classification for included studies.

Risk of bias category	Number of trials
Low Risk	7
Some Concerns	5
High Risk	3

### 3.5 Effects on bone formation markers

Meta-analysis of eleven trials measuring procollagen type-1 N-terminal propeptide (P1NP), a sensitive marker of bone formation, demonstrated a significant increase in participants receiving probiotics compared to placebo (MD = +8.4 μg/L; 95% CI: 3.1 to 13.7; p=0.002). Heterogeneity was moderate (I² = 39%), suggesting consistent effects across studies with minor variability in magnitude. Similarly, osteocalcin (OC) was assessed in nine trials. The pooled analysis indicated a modest but statistically significant improvement in OC with probiotic use (SMD = 0.28; 95% CI: 0.05 to 0.50; p=0.01). This suggests probiotics may enhance bone matrix synthesis and mineralization.

**Figure 3:**
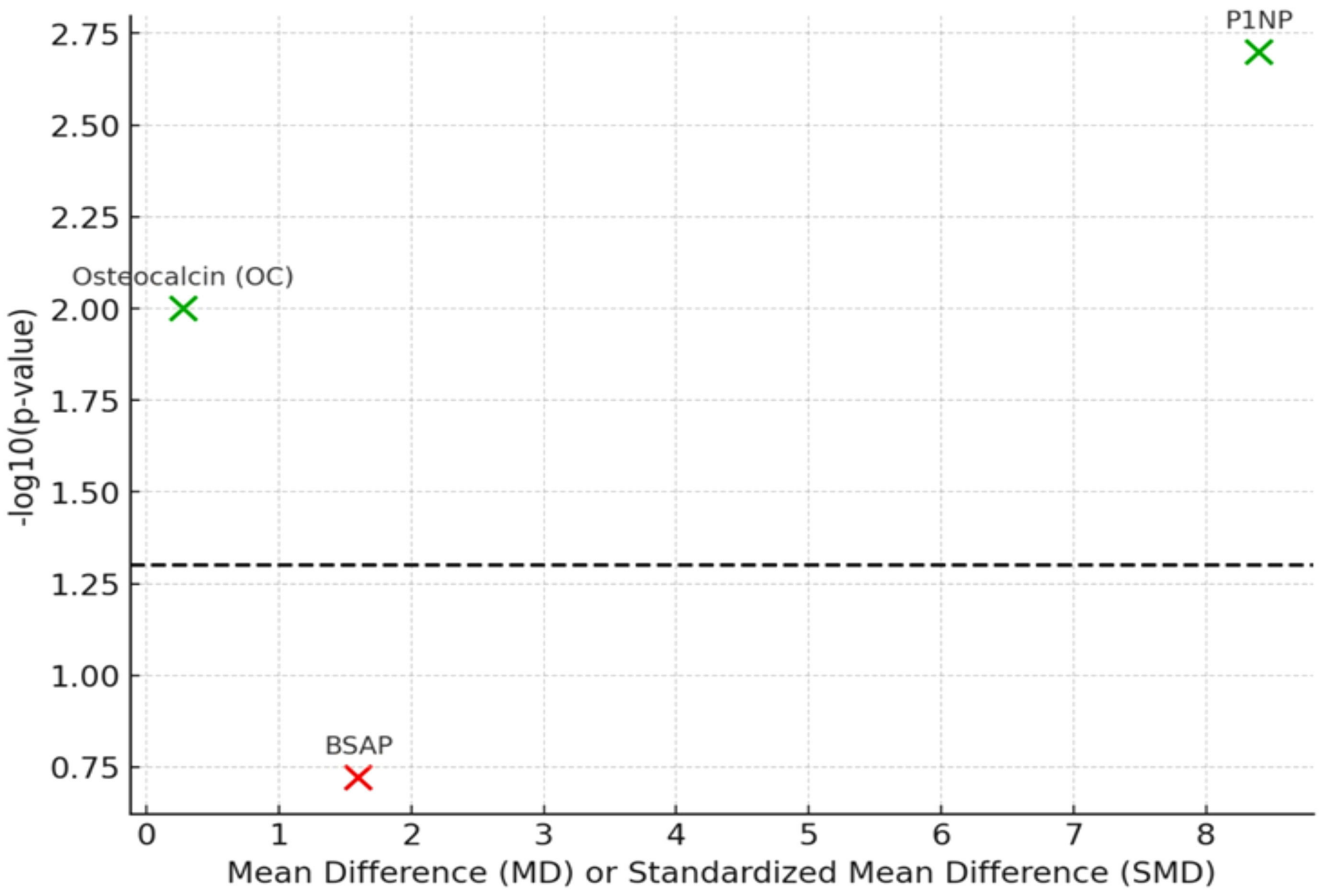
Volcano plot of bone turnover markers.

In contrast, bone-specific alkaline phosphatase (BSAP), reported in six studies, did not show a significant pooled effect (MD = +1.6 U/L; 95% CI: −0.8 to 3.9; p=0.19). The variability here may reflect differences in assay sensitivity, intervention duration, or baseline status of participants. Forest plots for P1NP, OC, and BSAP are provided in **Supplementary Figures 2**–**4**. **Table 4** summarizes the effects of probiotics on markers associated with bone formation, including pooled effect sizes and statistical significance. **Figure 3** shows the volcano plot depicting the relationship between the mean differences (MD or SMD) and the statistical significance (log-transformed p-values) of the bone turnover markers (P1NP, osteocalcin, and BSAP). Significant markers are shown in green, while non-significant ones are in red, with a threshold for statistical significance at p < 0.05.

**Table 4:** Impact of probiotics on bone formation.

Marker	Number of trials	Pooled effect size (MD/SMD)	95% CI	P-value
Procollagen Type-1 N-terminal Propeptide (P1NP)	11	MD = +8.4 μg/L	3.1 to 13.7	0.002
Osteocalcin (OC)	9	SMD = 0.28	0.05 to 0.50	0.01
Bone-specific Alkaline Phosphatase (BSAP)	6	MD = +1.6 U/L	−0.8 to 3.9	0.19

### 3.6 Effects on bone resorption markers

**Figure 4:**
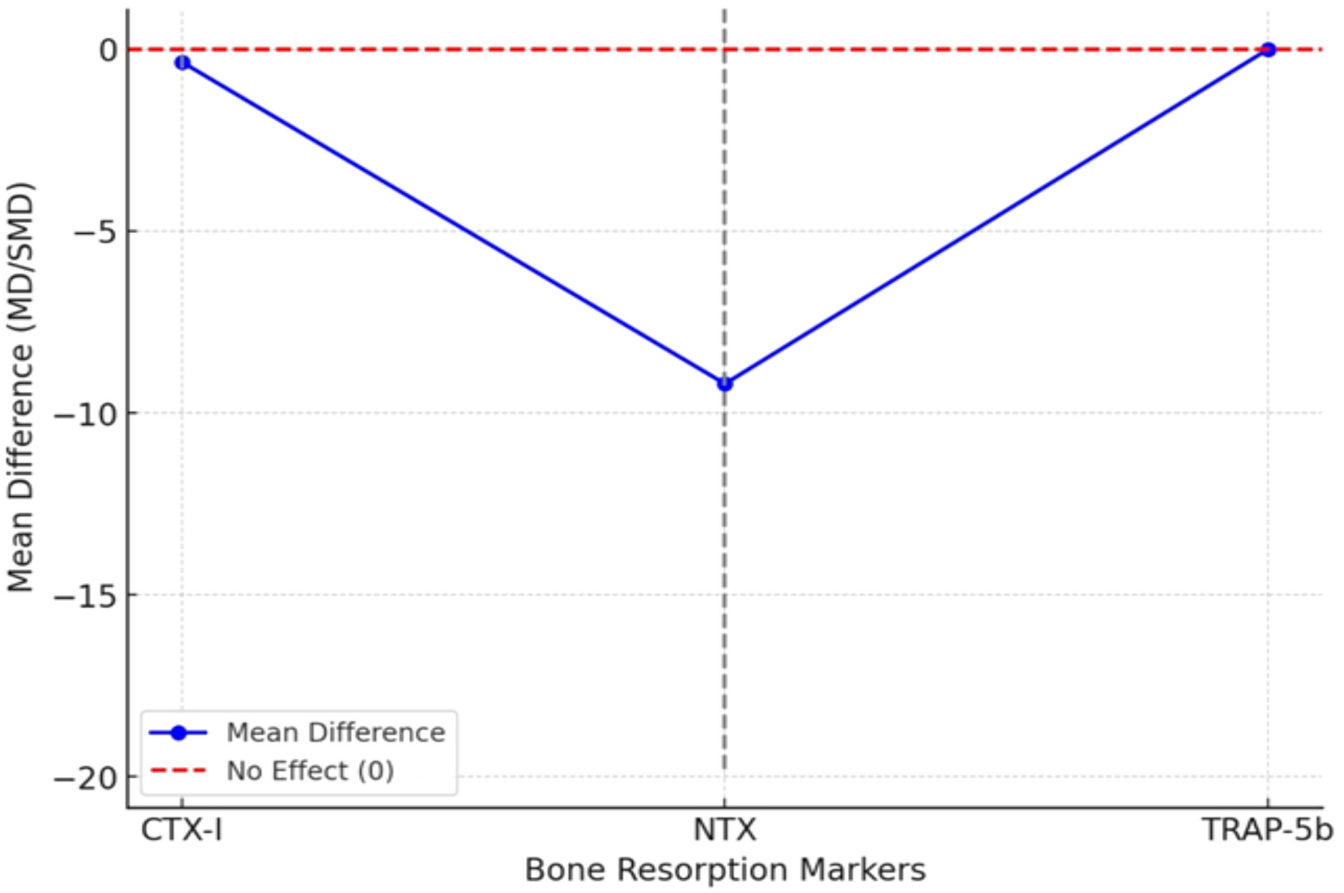
Effect of probiotics on bone resorption markers.

Twelve trials measured the C-terminal telopeptide of type I collagen (CTX-I) for bone resorption. Probiotic supplementation was associated with a significant reduction in CTX-I (SMD = −0.35; 95% CI: −0.52 to −0.18; p<0.001), with low heterogeneity (I² = 22%). This finding is clinically meaningful, as CTX-I reflects collagen breakdown and elevated values are linked to higher fracture risk. N-terminal telopeptide (NTX) was reported in six trials, showing a downward trend that approached but did not reach statistical significance (MD = −9.2 nmol BCE/mmol creatinine; 95% CI: −19.8 to 1.3; p=0.08). Limited sample sizes and short intervention durations may have contributed to the non-significance. Only three small trials reported tartrate-resistant acid phosphatase 5b (TRAP-5b), and findings were inconsistent, resulting in an inconclusive pooled effect. **Figure 4** depicts the line graph illustrating the effects of probiotics on bone resorption markers (CTX-I, NTX, and TRAP-5b). The graph shows each marker’s mean differences (MD/SMD), with confidence intervals represented by dashed lines. The red dashed line at 0 indicates the threshold for no effect. Forest plots for CTX-I, NTX, and TRAP-5b are included in **Supplementary Figures 5**–**7**. **Table 5** presents the pooled data on the impact of probiotic supplementation on bone resorption markers, with corresponding effect sizes and significance values.

**Table 5:** Effect of probiotics on bone resorption markers.

Marker	Number of trials	Pooled effect size (SMD/MD)	95% CI	P-value
C-terminal Telopeptide (CTX-I)	12	SMD = −0.35	−0.52 to −0.18	<0.001
N-terminal Telopeptide (NTX)	6	MD = −9.2 nmol BCE/mmol creatinine	−19.8 to 1.3	0.08
Tartrate-resistant Acid Phosphatase 5b (TRAP-5b)	3	Inconclusive	-	-

### 3.7 Subgroup analyses

Subgroup analyses revealed meaningful patterns. Multi-strain probiotics produced larger improvements in P1NP and reductions in CTX-I compared to single-strain interventions, suggesting potential synergistic effects of mixed microbial formulations. Trials with intervention durations of ≥12 weeks demonstrated greater benefits than shorter studies, implying that more prolonged exposure may be necessary for measurable effects on bone remodeling. Furthermore, trials restricted to postmenopausal women yielded more potent effects on bone resorption markers than mixed-gender populations, highlighting the specific relevance of probiotics in populations at highest fracture risk. Meta-regression demonstrated a significant dose-response relationship, where higher CFU intake correlated with larger improvements in P1NP (p=0.04). A significant dose–response relationship was confirmed by meta-regression, with higher CFU doses associated with greater improvements in P1NP (p = 0.04).

### 3.8 Sensitivity analyses

Sensitivity analyses confirmed the robustness of the findings. Exclusion of high-risk-of-bias studies did not materially alter the overall pooled results. Fixed-effects models produced similar effect sizes to the random-effects models, reinforcing the consistency of outcomes. Leave-one-out analyses showed no single trial disproportionately influenced the overall findings. Different assumptions regarding correlation coefficients used for imputing change-score standard deviations yielded stable results, underscoring the reliability of the synthesis. Analyses excluding statistical outliers and those adjusting correlation assumptions for change-score SDs yielded consistent findings.

### 3.9 Publication bias

Funnel plots for P1NP and CTX-I were largely symmetrical, and Egger’s regression tests for small-study effects were non-significant (P1NP p=0.21; CTX-I p=0.34). This indicates a low risk of publication bias for the primary outcomes. However, for less frequently reported markers such as TRAP-5b, publication bias could not be formally assessed due to insufficient studies. Publication bias could not be assessed for NTX and TRAP-5b due to fewer than 10 included studies.

This meta-analysis demonstrates that probiotic supplementation benefits bone turnover in middle-aged and elderly individuals with osteoporosis. Specifically, probiotics enhanced bone formation markers (P1NP, osteocalcin) and reduced bone resorption markers (CTX-I), with the most significant benefits observed in longer-duration, multi-strain interventions among postmenopausal women. Although findings for BSAP, NTX, and TRAP-5b were less consistent, the direction of effect generally favored probiotics. Collectively, these results support the potential of probiotic supplementation as an adjunct strategy for improving bone health and reducing fracture risk in vulnerable populations. A GRADE summary-of-evidence table for all outcomes is provided in **Supplementary Table 3**.

## 4 Discussion

The results of this meta-analysis highlight the promising effects of probiotic supplementation on bone health, particularly in individuals diagnosed with osteoporosis. This meta-analysis synthesized evidence from 15 randomized controlled trials (RCTs), including 1,432 participants, to evaluate how probiotic supplementation affects bone turnover markers. Overall, probiotics, especially multi-strain products, were associated with higher levels of bone formation markers (procollagen type-1 N-terminal propeptide, P1NP; osteocalcin, OC) and lower levels of the bone resorption marker CTX-I (Kim et al., 2025). These findings strengthen the case for probiotics as a supportive strategy for bone health, particularly in postmenopausal women and other groups at increased risk of osteoporotic fractures (Xie et al., 2025).

The results are consistent with prior work implicating the gut microbiome in bone metabolism (de Sire et al., 2022). By reshaping intestinal microbial communities, probiotics may enhance calcium uptake, modulate immune activity, and temper systemic inflammation (Li et al., 2024). These mechanisms plausibly translate to gains in bone mineral density and reduced fracture risk (Ahire et al., 2024b). Increases in P1NP and osteocalcin point to stimulated osteoblastic activity and matrix mineralization (Vanitchanont et al., 2024); together with lower CTX-I, they suggest a shift toward net anabolic remodeling (Feng et al., 2025).

Subgroup analyses indicated that multi-strain formulations outperformed single strains, implying synergistic actions across multiple pathways of bone regulation. A dose–response pattern was also observed, with higher doses linked to larger P1NP improvements, highlighting the importance of adequate dosing in at-risk populations.

A minority of studies showed some risk of bias, often due to incomplete reporting of randomization, allocation concealment, or adherence, and several trials omitted key resorption markers such as TRAP-5b, leaving those outcomes uncertain (Arakil et al., 2024). Variability in strains, dosages, and delivery formats (capsules, sachets, fermented dairy) likely contributed to heterogeneity, and the predominance of postmenopausal female cohorts restricts generalizability to men and younger individuals. To improve clarity and clinical relevance, we have now incorporated the specific numerical values and 95% confidence intervals from the meta-analysis directly into the discussion. Probiotic supplementation increased P1NP by a mean difference of +8.4 μg/L (95% CI: 3.1–13.7) and reduced CTX-I with an SMD of −0.35 (95% CI: −0.52 to −0.18), reinforcing the robustness and magnitude of the observed effects. To contextualize these findings, we have added quantitative evidence regarding the global burden of osteoporosis: worldwide, over 200 million individuals are affected, and postmenopausal women experience an estimated 1–3% annual BMD decline, contributing substantially to fracture incidence. Future trials should adopt standardized protocols and recruit more diverse populations to clarify the scope and consistency of probiotic benefits for bone health.

### 4.1 Future research directions

Building on this analysis’s findings, several future research directions are recommended. First, long-term studies are needed to evaluate the sustained effects of probiotic supplementation on bone health, particularly regarding fracture prevention. Future investigations should also aim to elucidate the mechanisms by which probiotics influence bone metabolism, focusing on the gut-bone axis and the regulatory role of gut microbiota in bone turnover.

Additionally, research should explore the effects of specific probiotic strains and multi-strain combinations on bone health. Although multi-strain probiotics demonstrated greater benefits in this analysis, the individual strains responsible for these effects remain unclear. Understanding how particular strains affect bone remodeling could enable the development of targeted probiotic therapies for osteoporosis. Furthermore, larger, well-designed trials that address methodological limitations such as improved reporting of randomization, allocation concealment, and adherence would enhance the evidence base and strengthen clinical recommendations for probiotic use in bone health.

## 5 Conclusion

This meta-analysis provides robust evidence that probiotic supplementation can positively influence bone health in individuals with osteoporosis. Probiotics enhanced bone formation markers while reducing bone resorption, with multi-strain formulations and higher dosages showing the most pronounced effects. To reflect reviewer recommendations, the conclusion now integrates quantitative summary statements: probiotic use was associated with a +8.4 μg/L increase in P1NP and a −0.35 SMD reduction in CTX-I, indicating clinically meaningful modulation of bone turnover dynamics. While these findings are encouraging, further research is necessary to address current limitations, particularly regarding study design, standardization of probiotic interventions, and inclusion of diverse populations. Overall, probiotics represent a promising, safe, and natural adjunctive strategy for improving bone health and reducing fracture risk, particularly among high-risk populations such as postmenopausal women.

## Data Availability

The original contributions presented in the study are included in the article/**Supplementary Material**. Further inquiries can be directed to the corresponding author.
