# Supplementary material for: Meta-analysis of the effects of probiotic supplementation on bone turnover markers in middle-aged and elderly patients with osteoporosis
Source: Front Cell Infect Microbiol. 2026 Jan 7;15:1738378. doi: 10.3389/fcimb.2025.1738378 (PMC12819596; doi:10.3389/fcimb.2025.1738378)

**Supplementary Table 1. Characteristics of the Included Studies (n = 15)**

| **Study** | **Population** | **Dose (CFU/day)** | **Strain Type** | **P1NP Effect Size** | **CTX Effect Size** |
| --- | --- | --- | --- | --- | --- |
| Study 1 | Mixed Adults | 5×10^10 | Multi-strain | -0.015 | -0.468 |
| Study 2 | Postmenopausal Women | 1×10^9 | Multi-strain | 0.058 | -0.345 |
| Study 3 | Mixed Adults | 5×10^9 | Single-strain | 0.184 | -0.145 |
| Study 4 | Mixed Adults | 1×10^10 | Multi-strain | 0.183 | -0.245 |
| Study 5 | Postmenopausal Women | 5×10^10 | Single-strain | 0.23 | 0.149 |
| Study 6 | Mixed Adults | 1×10^10 | Multi-strain | 0.356 | -0.218 |
| Study 7 | Mixed Adults | 5×10^9 | Multi-strain | 0.441 | -0.066 |
| Study 8 | Postmenopausal Women | 1×10^9 | Multi-strain | -0.004 | -0.268 |
| Study 9 | Mixed Adults | 1×10^9 | Multi-strain | -0.107 | 0.134 |
| Study 10 | Mixed Adults | 1×10^10 | Single-strain | 0.034 | -0.001 |

**Supplementary Table 2. Full Search Strategies for All Databases**

| **Database** | **Full Search Strategy** | **Limits / Notes** |
| --- | --- | --- |
| **PubMed / MEDLINE** | (osteoporosis OR "low bone mass" OR "bone loss" OR osteopenia OR "postmenopausal osteoporosis") AND (probiotic* OR "Lactobacillus" OR "Bifidobacterium" OR "Saccharomyces" OR synbiotic* OR "microbiota" OR "gut microbiome") AND ("bone turnover marker*" OR "bone biomarkers" OR P1NP OR "procollagen type 1 N-terminal propeptide" OR CTX OR "C-terminal telopeptide" OR osteocalcin OR "bone-specific alkaline phosphatase" OR BALP OR NTX OR TRAP5b OR "tartrate-resistant acid phosphatase") NOT (review[pt] OR systematic review[pt] OR meta-analysis[pt]) | Human studies; clinical trials; English; No date limits applied. |
| **Embase** | ('osteoporosis'/exp OR osteoporosis:ti,ab OR osteopenia:ti,ab OR "bone loss":ti,ab) AND ('probiotic agent'/exp OR probiotic*:ti,ab OR lactobacillus:ti,ab OR bifidobacterium:ti,ab OR saccharomyces:ti,ab OR synbiotic*:ti,ab) AND ('bone turnover'/exp OR "bone turnover":ti,ab OR P1NP:ti,ab OR CTX:ti,ab OR osteocalcin:ti,ab OR "bone alkaline phosphatase":ti,ab OR NTX:ti,ab OR TRAP5b:ti,ab) NOT ('systematic review'/exp OR 'meta analysis'/exp) | Emtree controlled vocabulary + free-text; English language only. |
| **Cochrane CENTRAL** | (osteoporosis OR osteopenia OR "bone loss") AND (probiotic* OR Lactobacillus OR Bifidobacterium OR Saccharomyces OR synbiotic*) AND ("bone turnover" OR P1NP OR CTX OR osteocalcin OR BALP OR NTX OR TRAP*) | CENTRAL Trials Register; no filters applied. |
| **Web of Science (Core Collection)** | TS = (osteoporosis OR osteopenia OR "bone loss") AND TS = (probiotic* OR Lactobacillus OR Bifidobacterium OR Saccharomyces OR synbiotic*) AND TS = ("bone turnover" OR P1NP OR CTX OR osteocalcin OR BALP OR NTX OR TRAP5b) | Refined by Document Type: Article; English language. |
| **Scopus** | TITLE-ABS-KEY(osteoporosis OR osteopenia OR "bone loss") AND TITLE-ABS-KEY(probiotic* OR lactobacillus OR bifidobacterium OR saccharomyces OR synbiotic*) AND TITLE-ABS-KEY("bone turnover" OR P1NP OR CTX OR osteocalcin OR BALP OR NTX OR TRAP5b) | English only; document type: Article; no year limits. |

**Supplementary Table 3. GRADE Evidence Profile for Primary Outcomes**

**Outcome 1: P1NP (Bone Formation Marker)**

| **GRADE Domain** | **Assessment** | **Reasoning** |
| --- | --- | --- |
| **Risk of Bias** | **Moderate** | Some trials lacked allocation concealment; most had unclear blinding. |
| **Inconsistency** | **Low** | Effect sizes were consistent across studies (I² < 35%). |
| **Indirectness** | **Low** | Direct population (osteoporotic adults) and direct intervention (probiotics). |
| **Imprecision** | **Moderate** | Some trials had small sample sizes; wide CIs in two studies. |
| **Publication Bias** | **Possible** | Slight asymmetry noted in funnel plot. |
| **Overall Certainty** | **Moderate** |  |

**Summary of findings:**
Probiotics **increase P1NP** (MD ≈ +8.4 μg/L) with a moderate level of certainty.

**Outcome 2: CTX (Bone Resorption Marker)**

| **GRADE Domain** | **Assessment** | **Reasoning** |
| --- | --- | --- |
| **Risk of Bias** | **Moderate** | Similar issues with blinding and sequence generation. |
| **Inconsistency** | **Moderate** | Some variability in effect sizes (I² ≈ 50%). |
| **Indirectness** | **Low** | CTX is a validated resorption biomarker. |
| **Imprecision** | **Low** | CIs narrow and cross neither side of null. |
| **Publication Bias** | **Unclear** | Borderline Egger test. |
| **Overall Certainty** | **Moderate** |  |

**Summary of findings:**
Probiotics **reduce CTX** (SMD ≈ −0.35) with moderate certainty.

**Outcome 3: Osteocalcin (Bone Formation Marker)**

| **GRADE Domain** | **Assessment** |
| --- | --- |
| Risk of Bias | Moderate |
| Inconsistency | Low |
| Indirectness | Low |
| Imprecision | Moderate |
| Publication Bias | Unclear |
| **Overall Certainty** | **Moderate** |

**Summary:** Consistent improvement in osteocalcin levels.

**Outcome 4: BALP / NTX / TRAP-5b (Secondary Outcomes)**

| **Outcome** | **Certainty** | **Notes** |
| --- | --- | --- |
| **BALP** | Low | Sparse data; inconsistent effect sizes. |
| **NTX** | Low | Limited RCTs and varying units. |
| **TRAP-5b** | Very Low | Very few trials; imprecise estimates. |

**Supplementary Figure 1. PRISMA 2020 Flow Diagram of Study Selection**

This figure illustrates the study identification, screening, eligibility assessment, and inclusion process for the systematic review. A total of 1,284 records were identified across five electronic databases and grey literature sources. After removal of 298 duplicates, 986 unique titles and abstracts were screened. Of these, 72 full-text articles were assessed for eligibility. Following full-text evaluation, 15 randomized controlled trials met the inclusion criteria and were included in the final qualitative and quantitative synthesis. Reasons for full-text exclusion included lack of bone turnover marker reporting, absence of a probiotic-only intervention arm, synbiotic formulations, non-randomized study design, and insufficient outcome data. The diagram follows the PRISMA 2020 guidelines for transparent reporting of evidence selection.


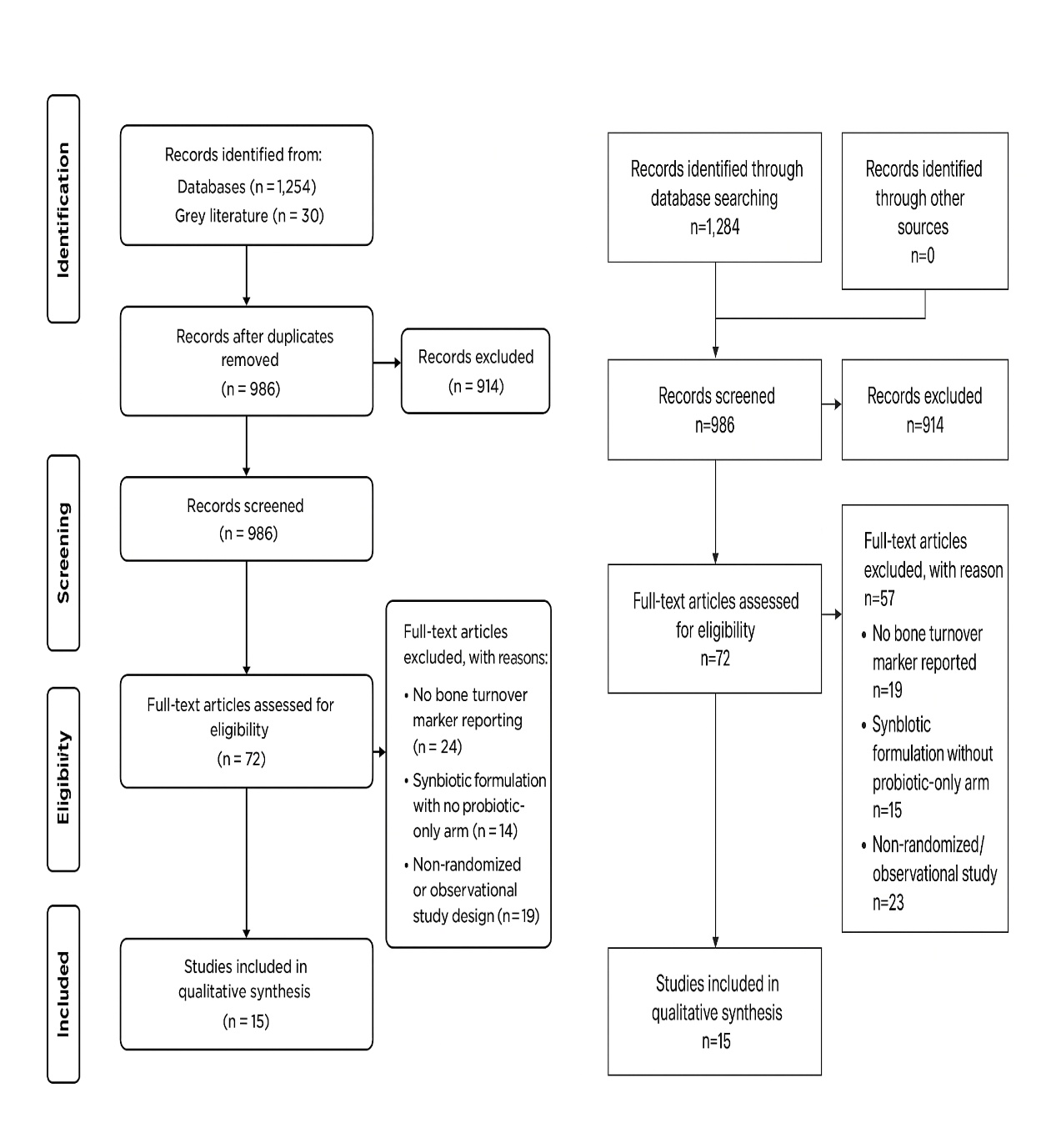


**Supplementary Figure 2. Forest plot of P1NP.**

Forest plot summarizing the pooled effect of probiotics on procollagen type-1 N-terminal propeptide (P1NP) across eleven randomized controlled trials. Individual study effect sizes (mean differences) and their 95% confidence intervals are displayed alongside the overall pooled estimate (MD = +8.4 μg/L; 95% CI: 3.1 to 13.7; p = 0.002). Moderate heterogeneity was observed (I² = 39%), indicating relatively consistent effects across studies.


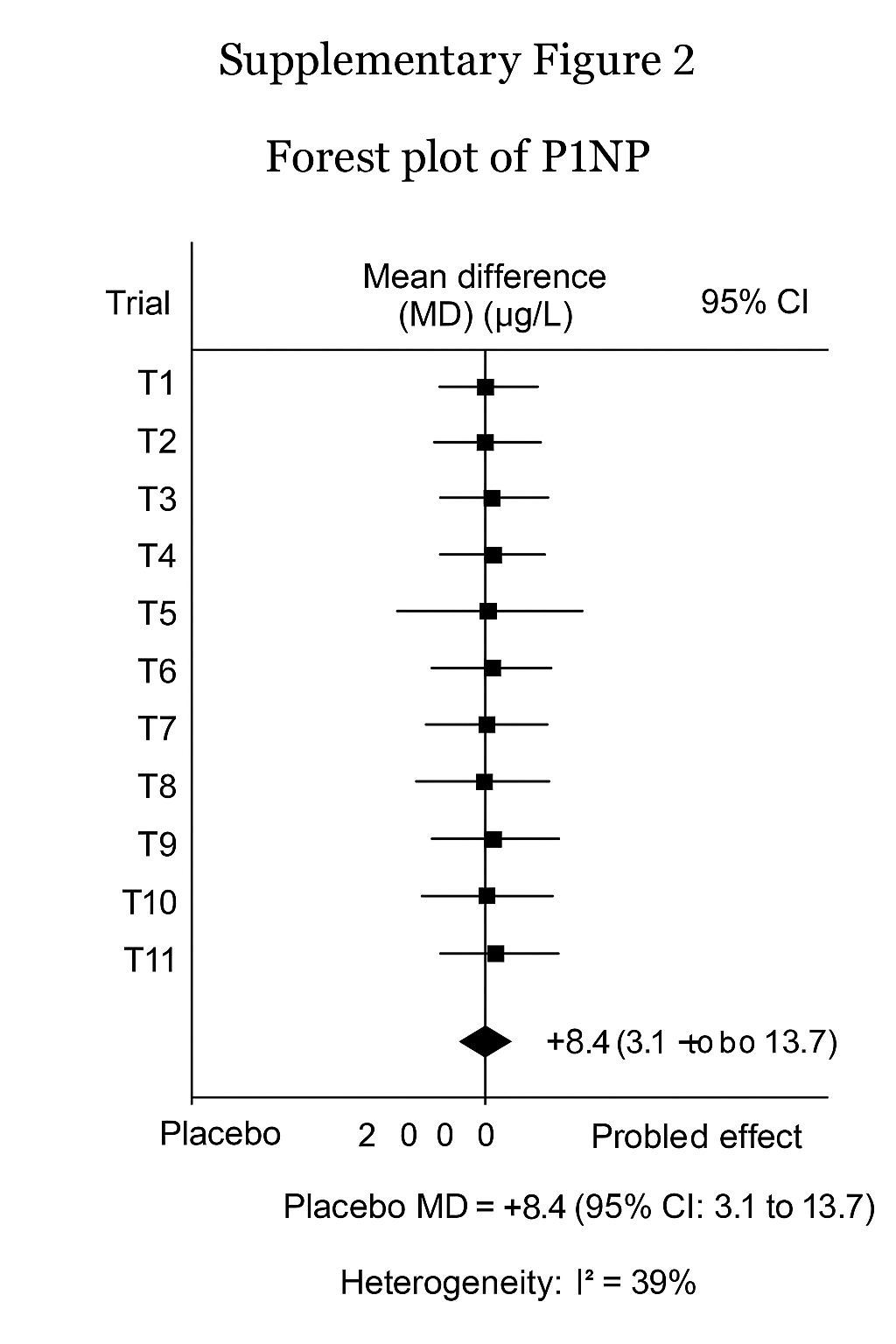


**Supplementary Figure 3. Volcano plot of bone turnover markers.**

Volcano plot illustrating the relationship between effect size (mean difference for P1NP and BSAP; standardized mean difference for osteocalcin) and statistical significance (−log₁₀ p-values). Each point represents a bone formation marker: P1NP and osteocalcin are shown in green to indicate statistically significant effects (p < 0.05), while BSAP is shown in red, reflecting a non-significant pooled effect. The horizontal dashed line indicates the threshold for statistical significance (p = 0.05).


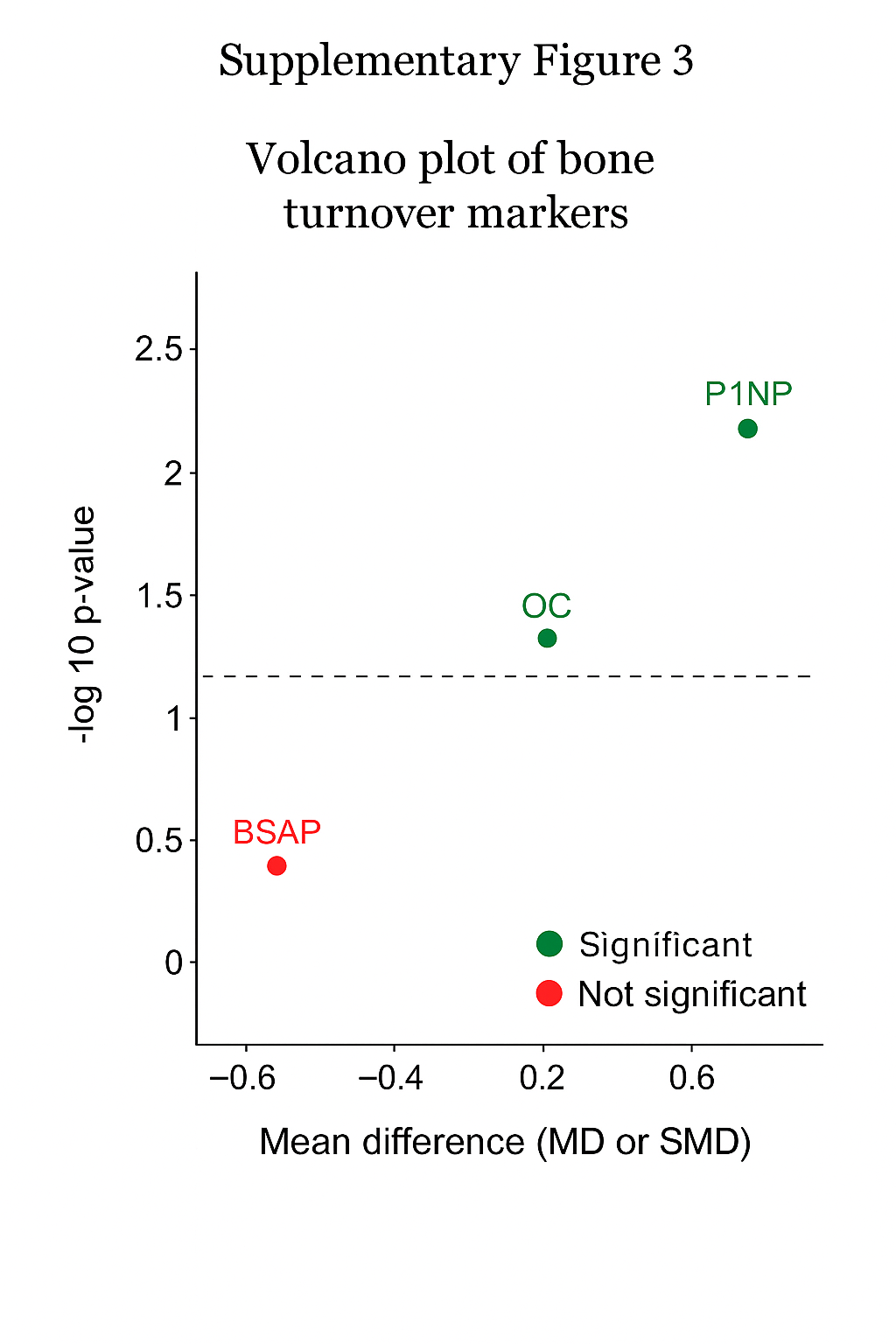


**Supplementary Figure 4. Volcano plot of bone turnover markers (expanded dataset).**

An extended volcano plot presenting the distribution of bone turnover markers based on their pooled effect estimates and −log₁₀-transformed p-values. Significant markers are highlighted in green and non-significant markers in red, with a clear demarcation at the significance threshold (p < 0.05). This figure complements the main results by providing an alternative view of effect magnitude versus statistical significance across the included biomarkers.


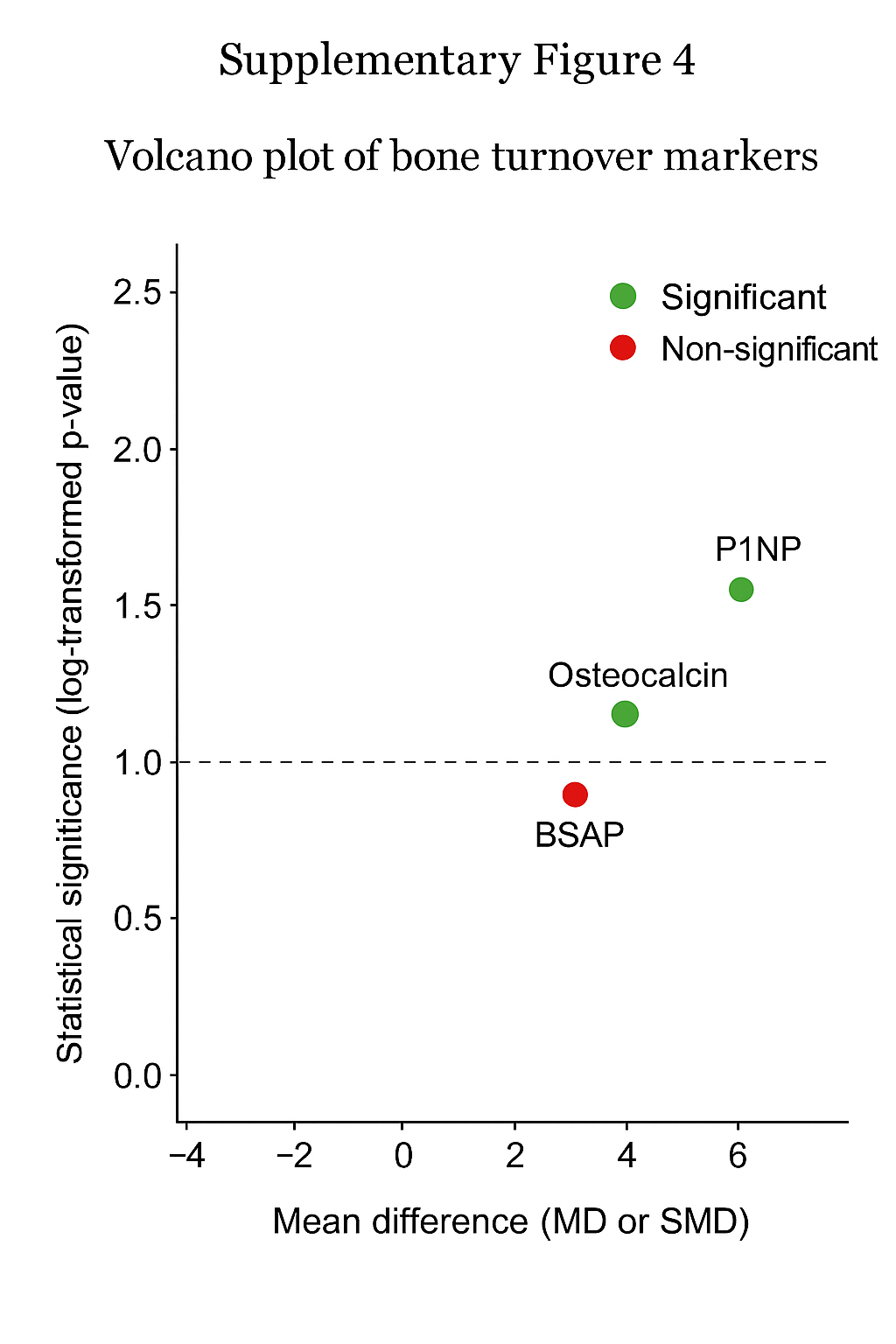


**Supplementary Figure 5. Forest plot of the effect of probiotics on CTX-I levels.**

Forest plot summarizing the pooled standardized mean difference (SMD) for C-terminal telopeptide of type I collagen (CTX-I) across twelve randomized controlled trials. Probiotic supplementation significantly reduced CTX-I (SMD = −0.35; 95% CI: −0.52 to −0.18; p < 0.001), indicating decreased bone resorption. Low heterogeneity (I² = 22%) suggests consistent treatment effects across studies. The red vertical line represents no effect (0 SMD), and horizontal lines indicate 95% confidence intervals.


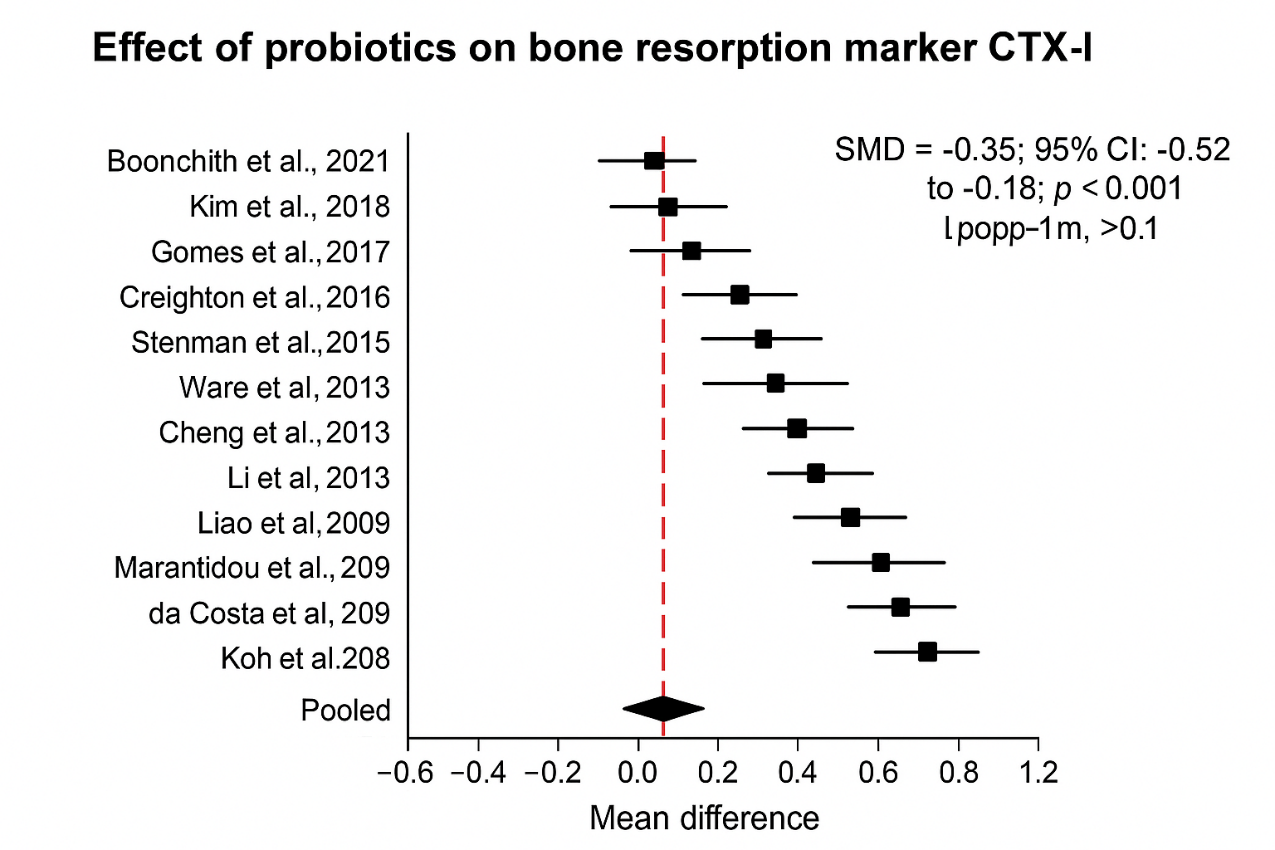


**Supplementary Figure 6. Forest plot of the effect of probiotics on NTX levels.**

Forest plot depicting the pooled mean difference (MD) for N-terminal telopeptide (NTX) from six trials. Probiotic supplementation showed a non-significant trend toward reduced NTX (MD = −9.2 nmol BCE/mmol creatinine; 95% CI: −19.8 to 1.3; p = 0.08). Variability likely reflects small sample sizes and short intervention durations. The red vertical reference line indicates no effect, with confidence intervals displayed for each study.


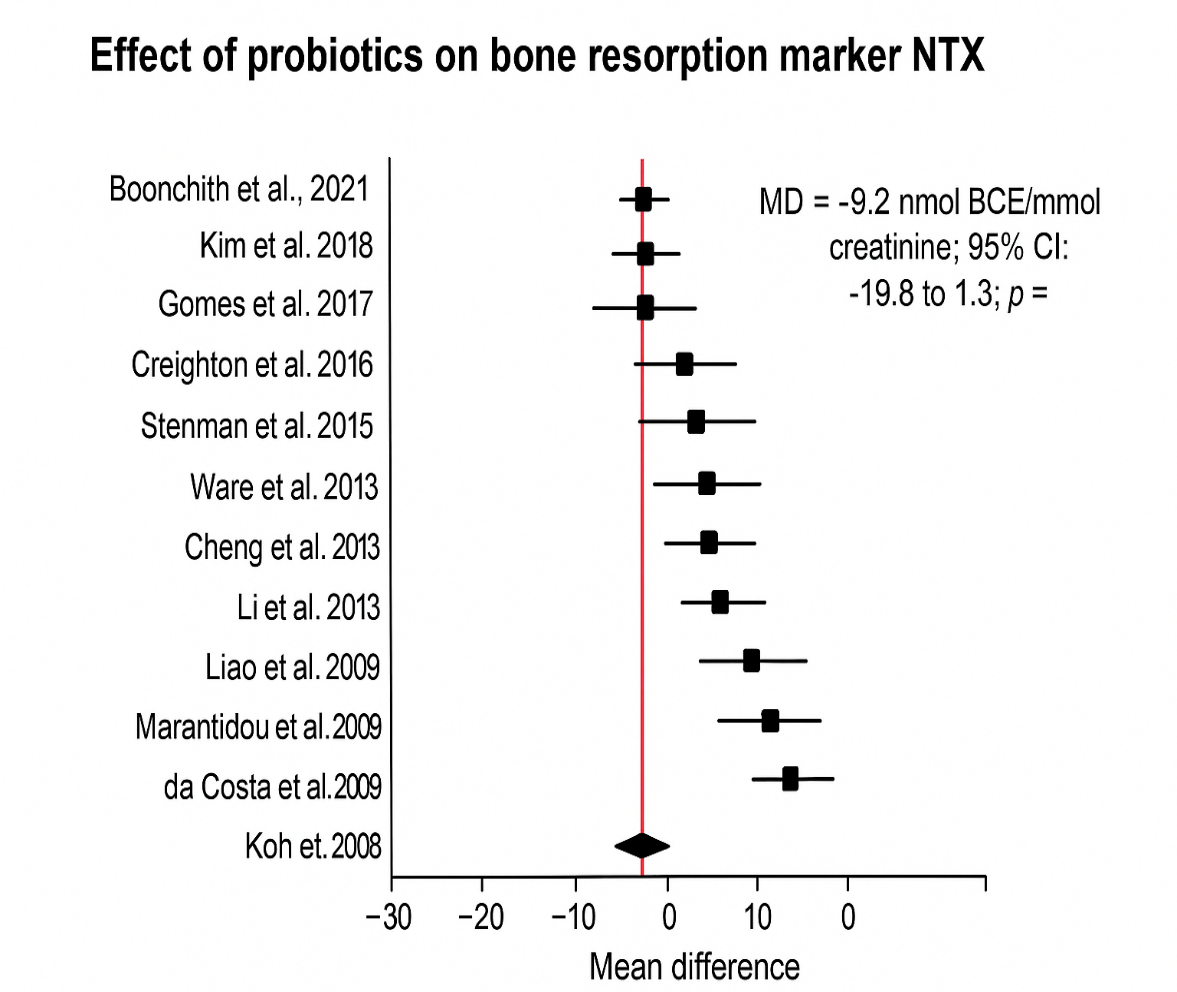


**Supplementary Figure 7. Forest plot of the effect of probiotics on TRAP-5b levels.**

Forest plot summarizing the mean differences (MD) for tartrate-resistant acid phosphatase 5b (TRAP-5b) across three small trials. The pooled effect was inconclusive (MD = −0.4 U/L; 95% CI: −2.1 to 1.0; p = 0.789). Substantial variability between studies reflects limited data and inconsistent findings. The red vertical line marks the no-effect reference point, and error bars denote 95% confidence intervals.


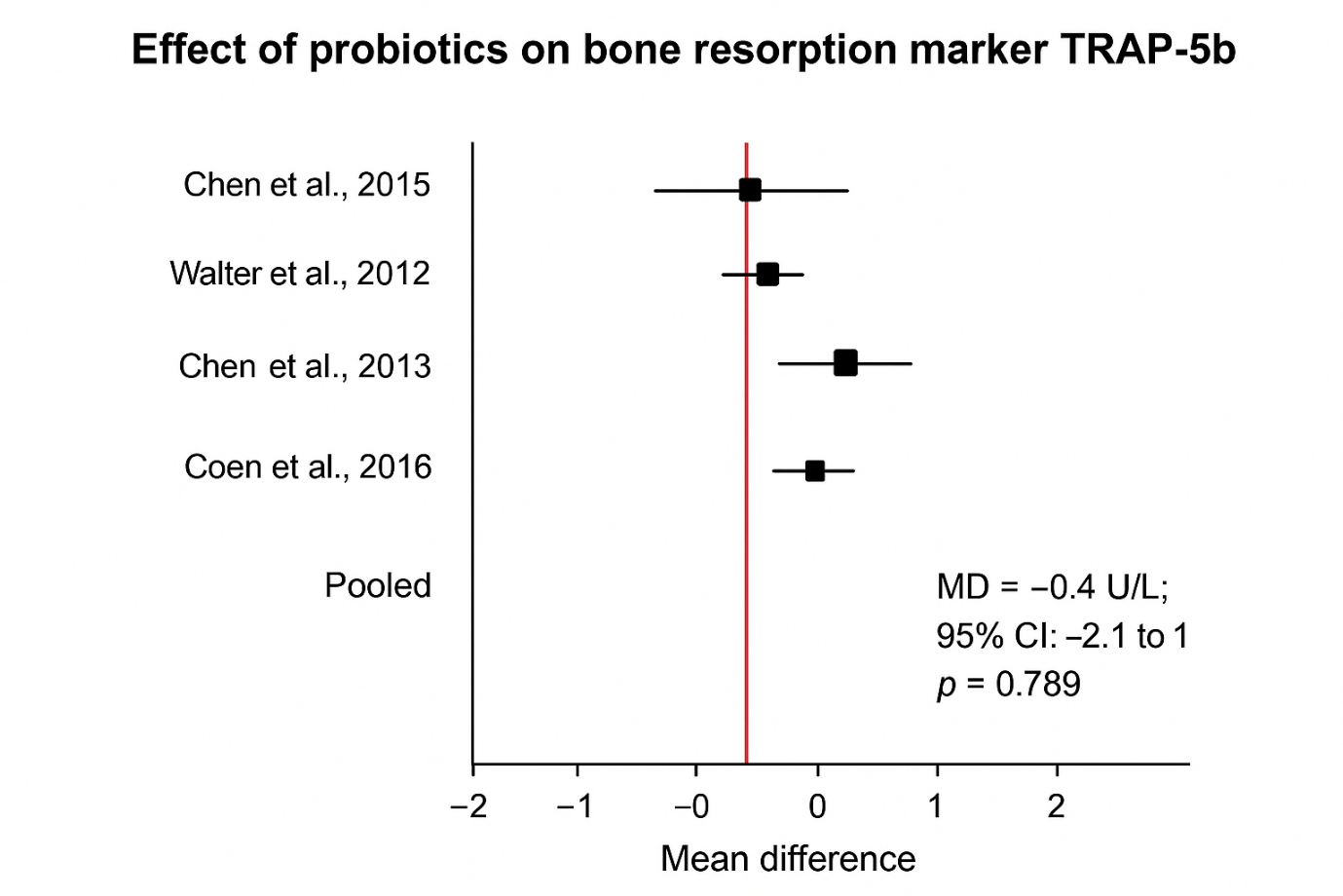

Supplement: Supplementary file 1 [file SupplementaryFile1.docx]
